# Supplementary material for: Chromothripsis during telomere crisis is independent of NHEJ, and consistent with a replicative origin
Source: Genome Res. 2019 May;29(5):737–49. doi: 10.1101/gr.240705.118 (PMC6499312; doi:10.1101/gr.240705.118)
Supplement: Supplemental Material [file supp_gr.240705.118_Supplemental_file_1.zip › contigs/annotated_contigs/DB84/contig.5.DB84_length_668_mean_cov_10.0988023952.docx]

**DB84_length_668_mean_cov_10.0988023952**

```
CTGTGTGTATATTTTCAAATGGCCACCTTAACAGAGGTAGTTAAAATTCCTATATTAAAGCACTTATTTTGAACAATAATGATCCTATA
 >chr2:57229880-57230258 + E=1e-209 p=1e-02
TAGATGGCACAAATATATATACACAATGTAATATTTCACAAGACATTCATAAAATATTTTAGAAATATTTTGGTGATACTTAAATTTTT

ACATATACGTGTTAAGAAATACTAAAATTTATTTAGCTCTATATAATGTTTATATTAAATATAACCATTATTTAAAGTGCCTGTGTGAT

TTTCTTGATTTCTTAAGCAAACATTGAGTATATACAATTTAAAATTTGCTTGATTCAAAAGTAATATAAAATGTATTGAGTAATTTATA

TAAAGAGAAGTGCTGGCATAAT|CATGAGAT|TGTGTATATATATATGTGTGTGTGTATACATATATATGGT|GT|GTGTATATATATG
                               >chr12:17389921-17389961 - E=3e-09 p=0e+00  >chr10:127
TGTGTGTATATATGGTATCTGTGTATAT|AC|ATGTATTCTGTTGTCATTGGATGGTATGTT|ATCTATATCT|ATATATATATAAACC
831726-127831767 + E=7e-07 p=2e-02  >chr18:41611308-41611338 - E=3e-03 p=0e+00  >chr2
TATGTTATATATATAAAACATATATATATATAAAACCAATGTTACATATATATATAGAACATACCACCCAACGGCAACAGAATACACAT
:57234083-57234240 + E=1e-79
TCTTCTCAAGTAAACATGGAATATTTTCCAGGATAGATCATATGTTAGCCCAT
```
